# The experience of setting up a resident-managed Acute Pain Service: a descriptive study

**DOI:** 10.1186/s12871-016-0179-0

**Published:** 2016-02-22

**Authors:** Tommaso Borracci, Daniela Prencipe, Anita Masotti, Alessandra Nella, Germana Tuccinardi, Lucia Margiacchi, Gianluca Villa, Fulvio Pinelli, Stefano Romagnoli, Angelo Raffaele De Gaudio, Giovanni Zagli

**Affiliations:** 1 Department of Anaesthesia and Intensive Care, Azienda Ospedaliero-Universitaria Careggi, Florence, Italy; 2 Department of Health Science, University of Florence, Florence, Italy

## Abstract

**Background:**

The benefits of an Acute Pain Service (APS) for pain management have been widely reported, but its diffusion is still limited. There are two APS models: anesthesiologist-based and a nurse-based model. Here we describe the development of a different APS model managed by anesthesia residents, and we report the first year of activity in a tertiary Italian university hospital (Careggi University Hospital, Florence, IT).

**Methods:**

Patients were included in the APS were those undergoing abdominal and urologic surgery causing moderate or severe postsurgical pain. The service was provided for patients, beginning upon their exit from the operating room, for 4, 12, 24 and 48 h for iv, and up to 72 h for epidural therapy. Vital signs, static/dynamic VAS, presence of nausea/vomiting, sedation level, and Bromage scale in case of epidural catheter, were monitored.

**Results:**

From September 2013 to April 2015, a total of 1054 patients who underwent major surgery were included in the APS: 542 from abdominal surgery and 512 from urological surgery. PCA and epidural analgesia were more adopted in general surgical patients than in urology (48 % vs 36 % and 15 % vs 2 %, respectively; *P* < 0.0001). Patients who underwent to abdominal surgery had a significantly higher self-administration of morphine (30.3 vs 22.7 mg; *P* = 0.0315). Elastomeric pump was the analgesic of choice in half of the urologic patients compared to a quarter of the general surgical patients (*P* < 0.0001). Among the different surgical techniques, epidural analgesia was used more in open (16.5 %) than in videolaparoscopic (1.9 %) and robotic technique (1.1 %), whereas PCA was predominant in videolaparoscopic (46.5 %) and robotic technique (55.5 %) than in open technique (31.4 %).

**Conclusions:**

The creation of APS, managed by anesthesia residents, may represent an alternative between specialist-based and nurse-based models.

## Background

Perioperative pain can be defined as pain present in surgical patients, caused by a pre-existing disease, the surgical procedure, or by the simultaneous presence of causes correlated with the surgery or pathology [1]. Since the first experience of treatment units for acute pain management (Acute Pain Service, APS) [2, 3], the benefits of a dedicated, multidisciplinary organization for pain management have been reported and accepted [4], even in terms of cost-effectiveness [5]. The Italian Society of Anesthesia Analgesia Resuscitation and Intensive Care Medicine (SIAARTI) promoted the standardization of procedures, the use of patient-controlled analgesia (PCA) and epidural analgesia (EA), and the development of APS for the adequate treatment of postoperative pain [1, 6]. Despite the existence in literature of guidelines for the management of postsurgical pain, its management still seem to be inadequate [7].

In a postoperative pain survey, Coluzzi and co-workers showed that postoperative pain continues to be under-managed in Italy: in a sample of 163 Italian hospitals (24.4 % of Italian public hospitals), only 41.7 % had an organized APS, most frequently in university and teaching hospitals [8]. In a second survey made in 2012, the sample included 289 hospitals (43.3 % of the Italian public hospitals), and results showed no substantial improvements since 2006 [9]. Thus, the correct management of post-operative pain is still a challenge in the Italian reality, and APS are not yet enough diffused.

It is possible to differentiate two main APS models: the first is the US model, which consists of anesthesiologist-based comprehensive pain management teams; the second is a nurse-based supervised APS, more diffused in European countries. Here we present the description of a different APS model and we report the first 12 months of activity.

## Methods

In September 2013, an APS for the post-operative period was organized in a tertiary Italian university hospital (Careggi University Hospital, Florence, IT). The management of APS was performed by residents of anesthesia.

The model adopted was based on residents in anesthesia, the “hub” of the system. Every day, a resident of anesthesia was dedicated exclusively to the APS service from 8 am to 8 pm. During the night, the Intensive Care Unit (ICU) resident on duty carried out the APS, assuring continuity of care. Patients included in APS were those undergoing abdominal and urologic surgery causing moderate or severe postsurgical pain. Operations were classified into major, intermediate, and minor, depending on the degree of pain that might be expected [10]. According to pain treatment guidelines, patients were assigned to a different treatment protocol based on surgery and health status. Patients were enrolled in APS if they showed moderate or severe postsurgical pain, or if the senior anesthesiologist was not completely sure of the adequacy of analgesia.

The service included the compilation of special forms for each patient at intervals of time, beginning upon exit from the operating room to 4, 12, 24 and 48 h for intravenous therapy, and up to 72 h for epidural therapy. Vital signs, static and dynamic VAS, presence of nausea/vomiting, sedation level, and Bromage scale in case of epidural catheter, were monitored. For the realization of our APS, we initially used a paper database, stored in a file cabinet. Beginning in January 2015, after modification and optimization of the initial model, a digital database was created, and a laptop (connected to the hospital server) was used to record bedside data. The patients’ records were created directly using a pc in the operating room, and the resident on duty in the APS received data on a laptop through the hospital Wi-Fi connection.

The protocol included the following therapies:

- Levobupivacaine 0.125 % + fentanyl 0.5 γ/ml, flow of 5 ml/h, patient-controlled bolus of 5 ml with a lock-out of 30 min;
- Elastomeric infusion pump (20 mg morphine diluted in 50 ml of saline solution, nominal flow of 2 ml/h);
- PCA: 0.5 mg/ml of morphine, patient-controlled bolus of 2 ml with a lock-out at 15 min, no continuous infusion;
- Scheduled iv repeated administration: paracetamol (0.5–1 gr every 6–8 h, maximum 4 gr/day);
- Rescue dose (if VAS was greater than 4): ketorolac 30 mg, maximum dose 90 mg/day.

This retrospective study was approved by the Institutional Ethics Committee of Careggi Hospital, which waived the need of informed consent for data publication due to the retrospective nature of the study and the anonymous data.

## Results

From September 2013 through April 2015, a total of 1054 patients who underwent major surgery were included in the APS: 542 from abdominal surgery and 512 from urological surgery. As summarized in Table 1, the percentage of open surgical technique was similar in abdominal and urological patients, whereas the robotic technique was predominant in urology, and laparoscopy was predominant in general surgery. Whereas open technique percentage was similar among general surgery and urology (45 % vs 47 %, respectively), percentage of videolaparoscopic technique resulted higher in general surgery (34 % vs 4 %; *P* < 0.0001); on the contrary, robotic surgery was used more in urologic patients than in general surgery (49 % vs 21 % respectively; *P* < 0.0001). Duration of surgery was similar among open (referral) and videolaparoscopic, whereas robotic technique resulted significantly longer (*P* = 0.0080) when compared with the referral technique (Table 1).

**Table 1:** Baseline characteristics of patients included in APS

	Overall	General surgery	Urology	*P*	Open surgery	Videolaparoscopic surgery	*P*	Robotic surgery	*P*
Number (%)	1054	542 (51.4 %)	512 (48.6 %)		484 (45.9 %)	206 (19.6 %)		364 (34.5 %)	
Age (years)	62.6 ± 14.8	62.5 ± 15.6	62.7 ± 13.9	0.8816	63.1 ± 14.9	64 ± 16.6	0.6394	61.1 ± 13.4	0.1544
BMI	25.6 ± 4.2	25.5 ± 4.8	25.8 ± 3.4	0.4678	25.8 ± 4.2	25.4 ± 4.2	0.3383	25.5 ± 4.3	0.4654
Gender (male), N (%)	640 (60.7 %)	264 (48.7 %)	376 (73.4 %)	**<0.0001**	320 (66.1 %)	90 (43.7 %)	**<0.0001**	230 (63.2 %)	0.3841
Duration of surgery (min)	265.2 ± 134	278.9 ± 137.4	250.2 ± 129.1	0.5363	264.4 ± 124.6	245.9 ± 139	0.2927	339 ± 147.3	**0.0080**

Videolaparoscopic and robotic techniques were compared to the open technique (referral)

Continuous data are expressed as mean ± standard deviation (SD). Statistical analysis: two-tailed Mann-Whitney test and two tails Fisher’s exact test. *P* significant if <0.05 (bold)

Table 2 summarizes the analgesic drug dosages and the devices used, an analysis based on general/urology and surgical techniques. Among general and urologic surgery, PCA and epidural analgesia were adopted more in general surgery than in urology (48 % vs 36 % and 15 % vs 2 %, respectively; *P* < 0.0001). On average, general surgical patients also had a significantly higher self-administration of morphine bolus than urologic patients (30.3 vs 22.7 mg; *P* = 0.0315). Elastomeric pump was the analgesic of choice in half of the urologic patients compared to a quarter of the general surgical patients (*P* < 0.0001). Among the different surgical techniques, epidural analgesia was used more in open (16.5 %) than in videolaparoscopic (1.9 %) and robotic technique (1.1 %), whereas PCA was predominant in videolaparoscopic (46.5 %) and robotic technique (55.5 %) compared to open technique (31.4 %) (Table 2).

**Table 2:** Analgesic drug dosages used in patients included in APS

	General surgery (*N* = 542)	Urology (*N* = 512)	*P*	Open surgery (*N* = 484)	Videolaparoscopic surgery (*N* = 206)	*P*	Robotic surgery (*N* = 364)	*P*
Intraoperative morphine (mg)	6.9 ± 2	6.6 ± 2.2	0.1613	6.7 ± 2.4	6.4 ± 2	0.4066	6.9 ± 1.8	0.2069
PCA, N (%)	262 (48.3 %)	186 (36.3 %)	**<0.0001**	152 (31.4 %)	94 (45.6 %)	**0.0005**	202 (55.5 %)	**<0.0001**
Patient-controlled morphine administration (mg)	30.3 ± 21.3	22.7 ± 20.7	**0.0315**	30.1 ± 27.9	35.6 ± 33.8	0.2928	20.2 ± 21	**0.0039**
Elastomeric pump, N (%)	140 (25.8 %)	258 (50.4 %)	**<0.0001**	196 (40.5 %)	64 (31.1 %)	**0.0205**	140 (38.5 %)	0.5708
Epidural catheter, N (%)	80 (14.8 %)	10 (2 %)	**<0.0001**	80 (16.5 %)	4 (1.9 %)	**<0.0001**	4 (1.1 %)	**<0.0001**
Scheduled iv repeated administration, N (%)	60 (11.1 %)	58 (11.3 %)	0.9223	56 (11.6 %)	44 (21.4 %)	**0.0013**	18 (4.9 %)	**0.0008**
Ketorolac (mg)	26.3 ± 9.6	24.7 ± 12.6	0.1985	24.6 ± 11	27.3 ± 12.8	0.1196	25.2 ± 10.5	0.6668
Paracetamol (gr)	1 ± 0.1	1 ± 0.1	0.3536	1 ± 0.2	1 ± 0.2	0.8980	1 ± 0.1	0.1386

Videolaparoscopic and robotic techniques were compared to the open technique (referral)

Continuous data are expressed as mean ± standard deviation (SD). Statistical analysis: two-tailed Mann-Whitney test and two tails Fisher’s exact test. *P* significant if <0.05 (bold)

**Fig. 1:**
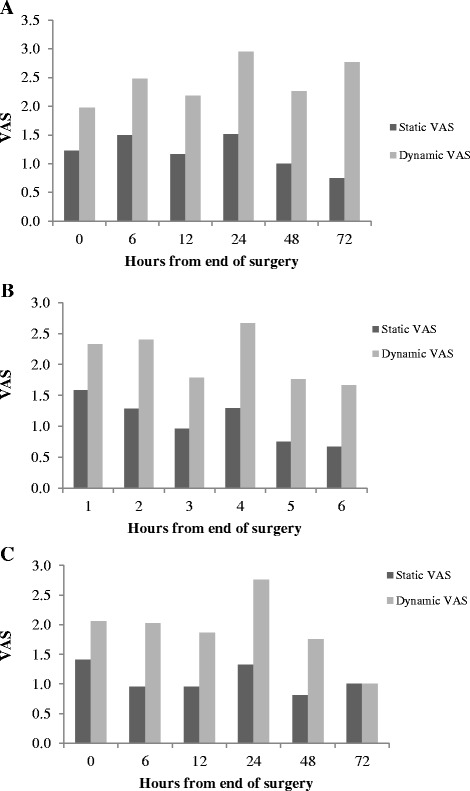
Comparison of static and dynamic VAS in patients who underwent to open (panel **a**), videolaparoscopic (panel **b**) and robotic technique (panel **c**)

**Fig. 2:**
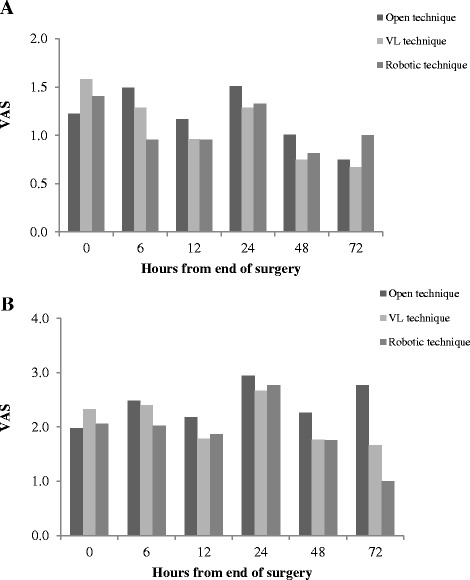
Comparison of static (panel **a**) and dynamic VAS (panel **b**) in open, videolaparoscopic and robotic surgery in patients who underwent to abdominal surgery

**Fig. 3:**
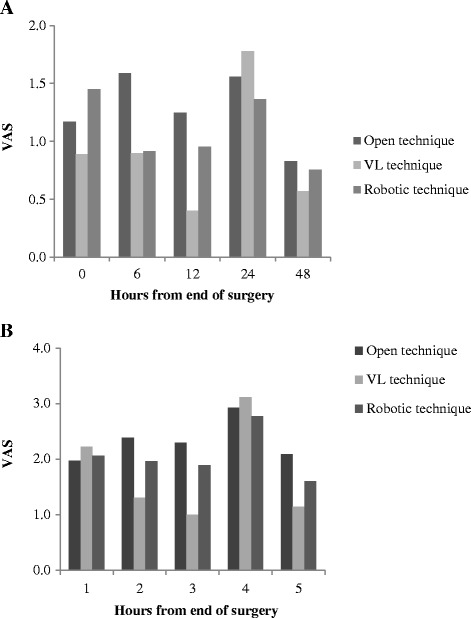
Comparison of static (panel **a**) and dynamic VAS (panel **b**) in open, videolaparoscopic and robotic surgery in patients who underwent to urologic surgery

As shown in Fig. 1, time course of VAS measurements showed a progressive reduction of level of pain. However, dynamic VAS remained significantly higher than the static VAS (*P* < 0.0001, Mann-Whitey test) in all three surgical techniques. The comparison between static and dynamic VAS in abdominal surgery (Fig. 2) and urological surgery (Fig. 3) showed that post-operative pain resulted similar among the different surgical techniques.

## Discussion

The creation of an APS permitted us to follow patients during the first 3 days after surgery, as well as to study and analyze the typology of patients, technique of analgesia, appropriateness of therapy. The recent survey of Coluzzi and co-Authors [9] underlined that post-operative pain is still under-treated, and confirmed that diffusion APS in Italy is still lower than other European countries [11]. Our results, when compared with POPSI-2 study [9], showed that the switch from elastomeric pump to PCA in our hospital can be considered satisfactory. However, even if our percentage of epidural catheters resulted acceptable compared with the data of Coluzzi and co-Workers, the finding that patients undergoing general surgery with open technique and managed with PCA self-administrated more morphine than urologic patients (Table 2), may suggest that we should increase the number of epidural catheters in general surgery. Notably, we observed that we did not use the as-needed analgesia modality anymore, which resulted to be highly prescribed in the survey (19 %) [9].

Concerning the different APS models, the US one (anesthesiologist-base) is effective but expensive, whereas the nurse-based supervised APS is a low-cost model in terms of the role of the anesthesiologist, which essentially is to teach and train nurses, supervise the APS, and select patients for special pain therapies [12]. Our model, managed by residents of anesthesia supervised by the senior anesthesiologist, could be considered a third choice, in which anesthesia residents, who have more professional skills than nurses, and can permit the early identification of patients who are developing post-operative complications (e.g. sepsis). In our experience, the ongoing development of an electronic database has represented an important progress in APS activity, as previously reported in a similar experience [13]. The use of a digital database, thereby updating data at the bedside with a laptop, permitted real-time analysis, data uniformity, and handover facilitation, in particular before the night shift.

Limitations of the present article must be discussed. First, as mentioned above, the lack of historical control group did not permit any comparison in terms of efficacy of analgesic therapy, appropriateness of devices used, complications rate. Second, we were not able to compare different model of APS organization, since this was our first experience. Finally, in consideration of the parameter used in pain evaluation (e.g. VAS), our sample size should be considered limited.

## Conclusions

The organization of APS should be a primary goal in post-operative care. Our experience suggests that the creation of APS model, managed by anesthesia residents, may represent an alternative between the US one (expensive, thus difficult to apply in Italian Healthcare System) and the nurse-based model.

## Abbreviations

APS: Acute Pain Service
ICU: Intensive Care Unit
PCA: Patient Controlled Analgesia
